# First-Line First? Trends in Thiazide Prescribing for Hypertensive Seniors

**DOI:** 10.1371/journal.pmed.0020080

**Published:** 2005-04-26

**Authors:** Steve Morgan, Kenneth L Bassett, James M Wright, Lixiang Yan

**Affiliations:** **1**Centre for Health Services and Policy Research, University of British ColumbiaVancouverCanada; **2**Pharmacology and Therapeutics Department, University of British ColumbiaVancouverCanada; University of SydneyAustralia

## Abstract

**Background:**

Evidence of reduced cardiovascular morbidity and mortality as well as cost support thiazide diuretics as the first-line choice for treatment of hypertension. The purpose of this study was to determine the proportion of senior hypertensives that received thiazide diuretics as first-line treatment, and to determine if cardiovascular and other potentially relevant comorbidities predict the choice of first-line therapy.

**Methods and Findings:**

British Columbia PharmaCare data were used to determine the cohort of seniors (residents aged 65 or older) who received their first reimbursed hypertension drug during the period 1993 to 2000. These individual records were linked to medical and hospital claims data using the British Columbia Linked Health Database to find the subset that had diagnoses indicating the presence of hypertension as well as cardiovascular and other relevant comorbidities. Rates of first-line thiazide prescribing as proportion of all first-line treatment were analysed, accounting for patient age, sex, overall clinical complexity, and potentially relevant comorbidities. For the period 1993 to 2000, 82,824 seniors who had diagnoses of hypertension were identified as new users of hypertension drugs. The overall rate at which thiazides were used as first-line treatment varied from 38% among senior hypertensives without any potentially relevant comorbidity to 9% among hypertensives with previous acute myocardial infarction. The rate of first-line thiazide diuretic prescribing for patients with and without potentially relevant comorbidities increased over the study period. Women were more likely than men, and older patients were more likely than younger, to receive first-line thiazide therapy.

**Conclusions:**

Findings indicate that first-line prescribing practices for hypertension are not consistent with the evidence from randomized control trials measuring morbidity and mortality. The health and financial cost of not selecting the most effective and least costly therapeutic options are significant.

## Abstract 2

Prescription practices for hypertension were not consistent with existing evidence--newer and more expensive drugs were prescribed preferentially despite evidence that thiazides are equally or more effective

## Introduction

Hypertension significantly increases the risk of serious cardiovascular morbidity and mortality and is the leading primary diagnosis for patient visits to physicians' offices in Canada [1]. Pharmaceuticals used in the treatment of hypertension constitute the leading therapeutic category of prescription drugs in Canada, accounting for 20% of total prescription drug sales in Canada [2]. Decisions concerning the management of hypertension will therefore have significant impact on population health and health care costs. First-line treatment of hypertension with thiazide diuretics has been shown to significantly reduce serious cardiovascular morbidity (stroke and myocardial infarction [MI]) and mortality in randomised controlled trials, with benefits at least as great as first-line treatment with other classes of antihypertensive drug [3,4,5,6]. Thiazide diuretics are also less costly than other antihypertensive drugs. Therefore, thiazide diuretics are the most cost-effective first-line therapeutic option for the majority of patients.

The purpose of this study was to determine whether prescribing practices are in accordance with this evidence. Using population-based research datasets at the University of British Columbia's Centre for Health Services and Policy Research, we calculated trends in first-line prescribing of antihypertensive drugs for seniors (residents aged 65 or older) in the province of British Columbia over the period of 1993 to 2000. We analyse the likelihood of first-line thiazide prescribing as a function of patient age, sex, overall clinical complexity, and potentially relevant comorbidities.

## Methods

Administrative data from public medical, hospital, and pharmaceutical insurance programs were analyzed to determine trends in first-line hypertension drug use. All residents of British Columbia are covered under a comprehensive public health insurance plan for medical and hospital services. Public insurance for prescription drugs is restricted to selected populations, but includes universal and comprehensive drug coverage for all seniors [7]. Administrative claims data from British Columbia PharmaCare, the public drug plan for all seniors, were used to track prescription drug use for this demographic cohort. Data from the public health insurance plan for medical and hospital services were used to identify diagnoses of hypertension and potentially influential comorbidities for those seniors who filled prescriptions for drugs commonly used in the management of hypertension.

The study cohort included all community-dwelling seniors who had evidence of first-time hypertension drug use and hypertension diagnosed in administrative databases. The classes of hypertension drugs were defined by the World Health Organization Anatomical Therapeutic Chemical classification system, and included angiotensin-converting enzyme (ACE) inhibitors, angiotensin-II receptor blockers, beta-blockers, calcium-channel blockers, alpha-antagonists, thiazide diuretics, nonthiazide diuretics, and other antihypertensives (e.g., reserpine). First-time hypertension drug use was defined as the receipt of any hypertension drug following at least one year of eligibility for PharmaCare coverage during which no prescriptions for any hypertension drugs were filled. Eligibility for coverage was measured starting in January 1992. A patient was considered a first-time user only once; those who stopped drug treatment and then filled prescriptions for antihypertensive drugs a year or more later were only included in the analysis of first-line use based on their initial course of treatment.

The study cohort was limited to the subset of first-time users of antihypertensive drugs who had a diagnosis of hypertension in administrative data records. This included patients for whom there was at least one diagnosis of hypertension (an International Classification of Diseases-9 code in the 401.x family) in Medical Services Plan and Hospital Separations databases spanning two years prior to the date of first-line hypertension treatment and one year following first-line treatment. Each Medical Services Plan record pertains to a fee-for-service billing and contains one diagnosis for the physician visit or related service. This is assumed the primary diagnosis for the visit or service. Hospital Separations records describe patient stays at the point of discharge and contain up to 16 diagnoses, all of which were searched. In order to avoid bias as a result of under-reporting the diagnosis of hypertension, diagnoses of hypertension were sought from records spanning two years prior to and one year following the first prescription for hypertension. Diagnoses occurring after drug use were included in the study because, for approximately one-third of users of hypertension drugs, the diagnosis of hypertension was recorded as the primary diagnosis during follow-up after the initiation of drug treatment.

Medical and hospital data spanning two years prior to the date of first-time hypertension treatment were searched for International Classification of Diseases-9 diagnostic codes pertaining to potentially relevant comorbidities. Identified using “Expanded Diagnostic Clusters” [8], the diagnoses sought included acute MI, aneurysm, angina, congestive heart failure, cardiac arrhythmia, cardiovascular valve disorder, gout, hyperlipidaemia, ischaemic heart disease, migraine headache, diabetes mellitus, and chronic renal failure. As has been done elsewhere [9], these comorbidities were selected because clinical guidelines, expert opinion, or marketing information suggested that their presence might influence the likelihood of a physician prescribing thiazide therapy; they are not necessarily substantiated by randomized controlled trial evidence of morbidity or mortality implications. A list of these diagnoses and their possible effects on treatment choices is provided in Table 1. Additional diagnoses, such as mild kidney disease, are also likely to be relevant, but these are not captured in administrative data.

**Table 1:**
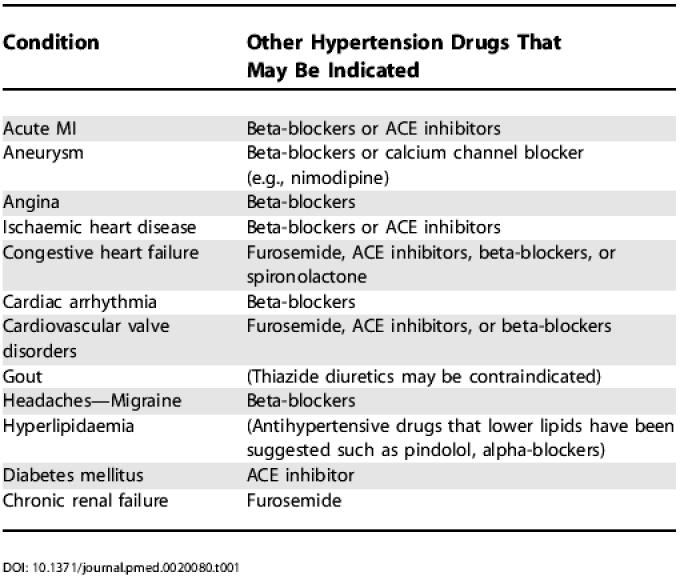
List of Potentially Confounding Conditions

Patients' first-line therapy was determined by the categories of drugs that they received on the date they first filled a hypertension prescription. In addition to patients who started treatment with the purchase of a thiazide alone or a combination product that contained thiazides, patients whose first hypertension treatment was a thiazide diuretic along with another antihypertensive drug were also considered to be receiving first-line thiazide therapy.

### Statistical Analysis

The analysis is based on data describing the entire population of users fitting the protocol definitions. Summary statistics describe average cost per day of treatment for each category of drug and first-line treatment choices over time. Multivariate logistic regression determined the probability that a patient would receive a thiazide diuretic as a first-line drug treatment. Independent variables for the multivariate analysis included patient age, sex, and year of first prescription. As a measure of overall clinical complexity, the regression included the number of different types of prescription drug purchased within the year prior to the patient's first antihypertensive drug purchase. Binary variables indicating prior diagnosis of comorbidities were included as independent variables. The analysis also included a binary variable that identified patients who filled no more antihypertensive prescriptions during the year following their first antihypertensive drug purchase. Wald chi-square tests were calculated to determine statistical significance.

## Results

A total of 82,824 British Columbia residents over age 65 received an antihypertensive drug for their first time between 1993 and 2000. Over the period of study, the share of British Columbia seniors who were first-time users of antihypertensive drugs in a given year increased from 1.8% to 2.2%. The median age of patient at the date of first-time hypertension use was 72; 55% of newly treated seniors were female.

**Figure 1:**
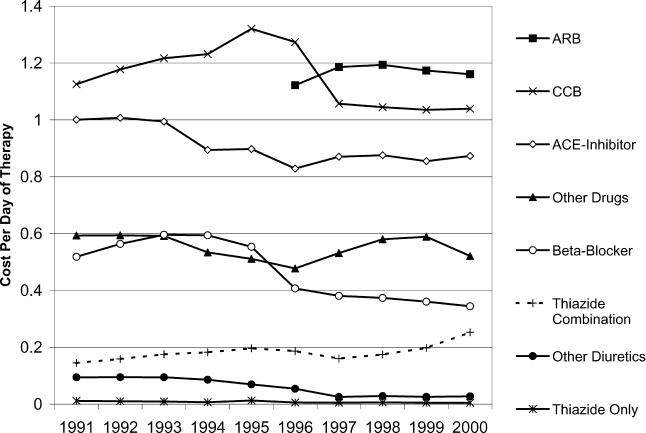
Average Cost per Day of Therapy Supplied to British Columbia Seniors by Category of Hypertension Drug

The average cost per days-supply of hypertension treatment received by British Columbia seniors varied by type of drug prescribed and was affected by policies implemented over the period of study (Figure 1). Available from 1996 onward, angiotensin-II receptor blockers were the most expensive treatment options by 2002, costing Can$1.16 per day of treatment. While the average cost per day of treatment with calcium-channel blockers had been as high as Can$1.32, it declined to Can$1.04 following the 1997 implementation of a reference-based pricing policy [10]. The daily cost of ACE inhibitors fell from Can$1.00 to Can$0.87 per day, also due to reference pricing [11]. Owing to increased generic availability, the cost per day of therapy on beta-blockers fell from Can$0.59 to Can$0.34 over the period. Over the entire period, thiazide diuretics were the least expensive treatment option, costing less than Can$0.01 per day of therapy.

**Figure 2:**
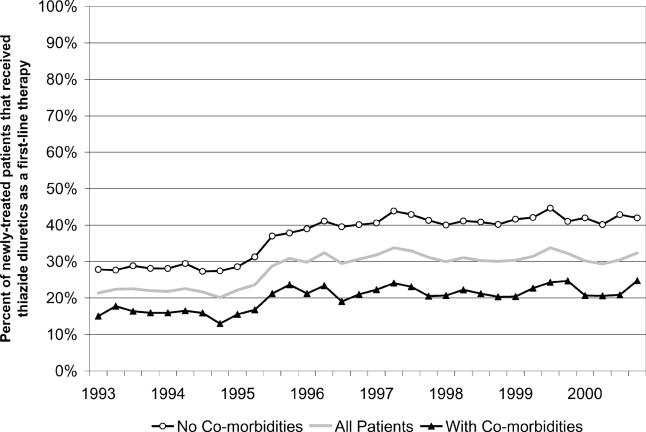
Thiazide Diuretics as Percentage of First-Line Treatments for Hypertensive Patients with and without Potentially Confounding Comorbidities

Thiazide diuretics were prescribed as a first-line therapy for less than a third (29%) of newly treated patients. Over time, the trend in the share of newly treated patients that received thiazide diuretics as a first-line therapy was similar for those with and without diagnoses indicating potentially influential comorbidities. Figure 2 illustrates these time trends. The shares of patients with and without comorbidity that received first-line thiazide treatment increased from 25% and 16%, respectively, in 1995 to 42% and 23% in 1997. These shares remained relatively stable thereafter, ending at 42% and 22% in 2000. First-line thiazide diuretic prescribing as a share of newly treated patients did not exceed 45% at any point from 1993 to 2000.

The proportion of newly treated patients receiving thiazides as first-line therapy varied according to the presence of comorbidity diagnoses (Table 2). Nearly half (39,764) of these newly treated senior hypertensives had no medical or hospital records indicating a potentially confounding comorbidity; 38% of these “uncomplicated” patients received thiazides as first-line treatment. The 22% of newly treated patients that had multiple comorbidities received thiazides at a rate of approximately 1 in 7 (14%). Those with evidence of previous acute myocardial infarction received thiazides least frequently (9%). As shown in Table 3, multivariate logistical regression analysis indicated that first-line treatment was affected by a time trend (rates of thiazide use increased over the period, *p* < 0.001), gender (women more likely to receive thiazides, *p* < 0.001), and age (older patients more likely to receive thiazides, *p* < 0.001). The measure of overall clinical complexity (number of different drugs used) did not have a significant impact. After adjusting for these factors, all potentially influential concurrent diagnoses other than migraine headache were associated with a statistically significant reduction in the likelihood of first-line thiazide diuretic use. Patients with diagnoses of acute MI, angina, or chronic renal failure were less than half as likely to receive thiazide diuretics as those without such diagnoses.

**Table 2:**
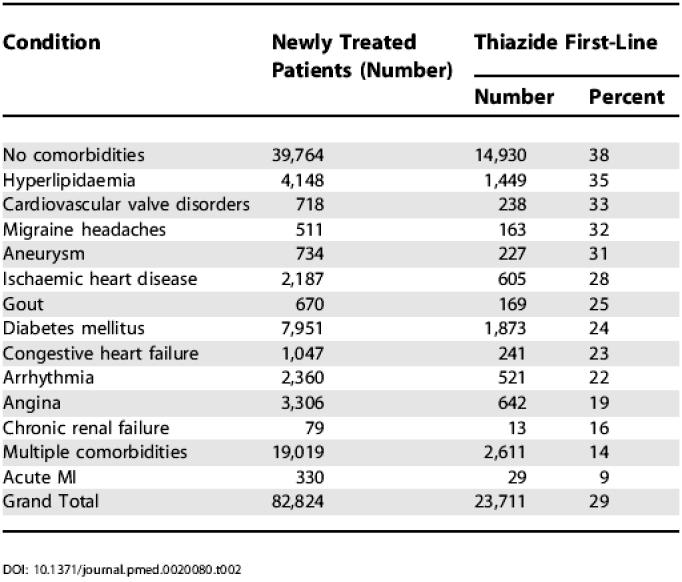
Total Newly Treated Patients and Rate of First-Line Thiazide Use by Comorbidity

**Table 3:**
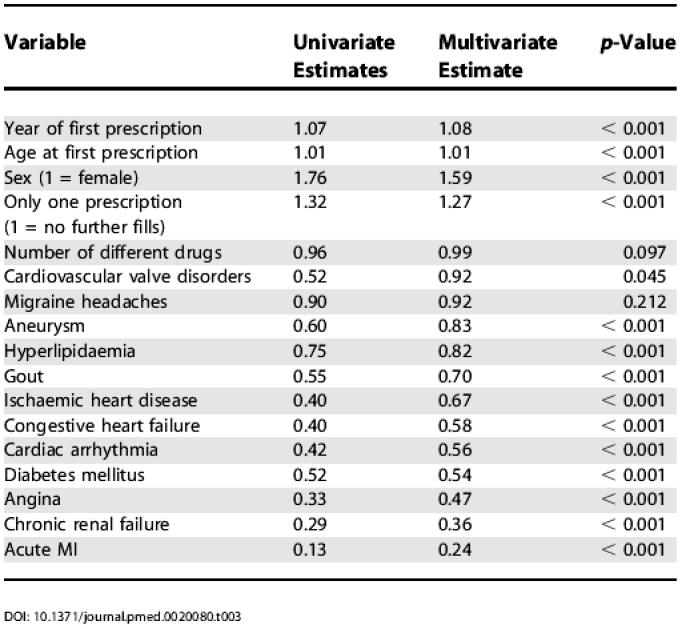
Logistic Regression Results—Odds Ratios

### Study Limitations

Despite the advantage that it is population based, this research study is limited by the use of administrative claims data. Claims data may result in under-reporting of actual conditions [12,13]. In an effort to compensate for this, the definitions of comorbidities were based on one or more diagnoses over a two-year period. A further limitation of claims-based data is that they track only those prescriptions that are dispensed from pharmacies. This analysis therefore does not track first-line use by way of professional samples given to patients. Because thiazide diuretics are no longer patented, none of their manufactures provide professional samples in British Columbia. Thus, the results may over-report the true first-line use of thiazides.

## Discussion

The overall share of British Columbia senior hypertensives that received thiazide diuretics as first-line treatment ranged from 21% to 34% between 1993 and 2000. Thiazide use was affected by the presence of diagnoses for certain comorbidities; however, the rates of first-line thiazide use were relatively low (below 45%) for patients both with and without any evidence of conditions that might influence treatment choice. We believe that such rates of first-line thiazide use among newly treated hypertensive seniors are low, given the evidence for the morbidity and mortality benefit associated with the first-line use of this class of antihypertensive drugs [4,5,6,14] and the substantial cost differences between products.

Because the cost per day of treatment on alternative treatments is upwards of 100 times that of thiazide diuretics, the financial consequences of first-line treatment decisions are significant, even when limited to the group of “uncomplicated” cases. For the subset of patients with no comorbidities, the additional cost to British Columbia PharmaCare in 2000 stemming from decisions to treat newly hypertensive seniors with nonthiazides was approximately Can$1 million per year. This additional cost will accumulate for each year that these incident cases continue to receive treatment. It is therefore necessary for professional associations and those who pay for (and benefit from) pharmaceutical benefits to work together to ensure that prescribing practices more accurately reflect research evidence.

One might conjecture that low rates of first-line thiazide use result from physicians' concerns regarding the presence of comorbidity. In our study, approximately half of the newly treated hypertensive seniors had diagnoses associated with specific comorbidities that may influence prescribers' beliefs about the appropriateness of thiazide diuretics. In all instances except migraine headaches, the presence of a comorbidity diagnosis predicted a decreased incidence of first-line thiazide use. Some of the associations are rational and consistent with outcomes evidence. Following acute MI, for example, beta-blockers and ACE-inhibitors are first-line choices because they have been shown in randomized controlled trials to reduce morbidity and mortality in addition to having a blood pressure lowering effect [15,16]. It is encouraging that in accordance with the evidence in this population, we found that only 9% of patients received thiazide diuretics as first-line therapy. Some of the associations that we found between comorbidity and first-line antihypertensive choice were not substantiated by evidence of morbidity and mortality implications. Hyperlipidaemia, for example, was associated with a statistically significant decrease in thiazide use versus patients without this diagnosis. This is likely explained based on the reported small increase in cholesterol associated with thiazides [17], but is not rational based on the morbidity and mortality evidence from randomized controlled trials [5,6,17].

Clinical practice guidelines starting in the early 1980s recognized the benefits of treatment with thiazide diuretics by recommending them as the first-line treatment of choice for virtually all hypertension patients [18,19,20,21,22,23,24,25]. Newer guidelines, while acknowledging thiazide diuretics as a first-line choice, increasingly recommended other classes of antihypertensive drugs as “alternate” first-line choices or recommended other classes for specific patient populations [26,27,28]. An example of this is the recommendation against use of thiazide diuretics for patients with diabetes mellitus. It was argued that thiazide diuretics might not achieve the same morbidity and mortality benefits because they increase blood glucose in some patients. However, this effect of thiazides on glucose has not diminished the morbidity and mortality benefit seen in randomized controlled trials: patients with diabetes achieve similar morbidity and mortality benefit with thiazides to non-diabetic patients [29]. Similarly, recent clinical practice guidelines recommend ACE-inhibitors as the preferred first-line therapy for hypertensive patients with mild kidney disease [28]. Proponents of ACE-inhibitors argue that hypertensive patients with kidney disease benefit both from the general antihypertensive effect of ACE-inhibitors and a specific 'kidney sparing' effect associated with this class of drugs. This theoretical benefit has not been substantiated in randomized controlled trials [30].

Given that hypertension treatment is one of the fastest growing drivers of seniors' drug expenditure in British Columbia [31], a critical challenge facing clinicians and policy makers is to use lower-cost therapeutic options whenever they produce equivalent or superior morbidity and mortality benefit to patients. This can be a particularly difficult task when set against a backdrop of marketing claims that draw associations between marketed products and superior performance for using surrogate markers for sub-populations. One mechanism for improving the cost-effectiveness of product selection decision is education. Our study results show that first-line thiazide therapy increased towards the end of 1995 and through 1996, and then remained at a relatively stable rate of approximately 30%. The increase in the rate of first-line thiazide prescribing coincided with the publication of two Therapeutics Letters, produced by the Therapeutics Initiative at the University of British Columbia, on the topic of first-line hypertension treatment [4,14]. The observed impact of these letters, which were mailed to all prescribing physicians in British Columbia in the summer and fall of 1995, has been reported elsewhere [9,32]. Except for this increase, the rate of first-line thiazide therapy did not change substantially over the study period despite a growing body of available evidence indicating the morbidity and mortality benefit of first-line thiazide therapy [5,6,33,34]. Policymakers might consider engaging in regularly reinforced educational interventions to better inform prescribers regarding the appropriate cases for selecting lower and higher cost medicines. Alternatively, or perhaps in conjunction with educational efforts, innovative forms of incentive pricing or benefit-sharing may be used to provide both prescribers and patients the incentive to start with the low cost therapies [35]. Either way, it is incumbent upon the medical profession to weigh carefully the relative costs and benefits of treatments for particular patients; for, every additional dollar spent on drugs without any benefit is a dollar that is unavailable for other medical or pharmaceutical services.

This analysis predates publication of the largest randomized antihypertensive trial designed to answer the question of which first-line antihypertensive is best, the ALLHAT trial [30]. ALLHAT compared four first-line antihypertensive classes (a thiazide-like diuretic [THZ], an alpha blocker, an ACE inhibitor, and a calcium channel blocker) in 33,357 patients. The alpha-blocker caused increased cardiovascular morbidity, and that arm was terminated early [36]. At the end of the trial, total mortality, coronary heart disease, and end-stage renal disease were similar for the remaining three arms. The first-line calcium channel blocker caused more heart failure compared to the THZ and ACE inhibitor. First-line ACE inhibitor caused more strokes compared to THZ. The overall conclusion from ALLHAT was that thiazide diuretics are the preferred first-line therapy for hypertension [30].

**Patient Summary**

**Background**

High blood pressure (hypertension) is common and increases the chances of developing heart disease. Different types of drugs are available for treating blood pressure, and they have been compared in many different studies. When doctors recommend a particular drug for a particular patient, they base their decision on the latest studies, on whether the patient has any other medical problems that might affect drug choice, and on the price differences between different drugs. If there are two drugs that have been shown to work equally well for a particular patient, the doctor should prescribe the cheaper one.

**Why Was This Study Done?**

The researchers wanted to see whether doctors follow the latest guidelines when they prescribe drugs for treating high blood pressure (antihypertensive drugs).

**What Did the Researchers Do?**

They looked at which drugs doctors had given to people aged over 65 years with high blood pressure in British Columbia, Canada, from 1993 to 2000. They only looked at prescriptions for people who had never taken blood-pressure medication before, a total of 82,824 patients. The drug of choice for most of these patients is a thiazide. Thiazides belong to a class of drugs called diuretics, sometimes referred to as water pills. These drugs have been around for a long time and in most patients they are at least as effective as and much cheaper than any of the newer drugs.

**What Did They Find?**

The prescription of thiazides in these patients increased from 22% to 33% during the study period, but it was consistently lower than it should have been based on the existing evidence.

**What Does This Mean?**

Doctors in British Columbia do not prescribe thiazides for seniors with high blood pressure as much as they should. As a consequence, some patients don't get the best treatment and the costs are considerably higher than they need to be. The reasons are not clear, but the findings suggest that doctors might not be up-to-date with the latest study results and guidelines. There have been more studies since 2002 and more efforts to educate doctors, and it would be interesting to know whether the current prescription practices better match the latest results.

**More Information Online**

The website of the US National Heart, Lung, and Blood Institute (http://www.nhlbi.nih.gov) has information about antihypertensive drugs and their characteristics, including facts on a recent big study (called the ALLHAT study) that compared antihypertensive drugs: http://www.nhlbi.nih.gov/health/allhat/facts.htm

Information pages from the Blood Pressure Association, a UK-based charity: http://www.bpassoc.org.uk/information/information.htm

Medline Plus information pages on blood pressure: http://www.nlm.nih.gov/medlineplus/highbloodpressure.html

## Abbreviations

ACE: angiotensin-converting enzyme
MI: myocardial infarction
THZ: thiazide-like diuretic
